# Preferred HIV testing services and programme characteristics among clients of a rapid HIV testing programme

**DOI:** 10.1186/1471-2458-13-791

**Published:** 2013-08-30

**Authors:** Juan Hoyos, María José Belza, Sonia Fernández-Balbuena, María Elena Rosales-Statkus, José Pulido, Luis de la Fuente

**Affiliations:** 1 Carlos III Health Institute, National Epidemiology Centre, C/ Monforte de Lemos, nº5, 28029 Madrid, Spain; 2 CIBER Epidemiology and Public Health (CIBERESP), C/Monforte de Lemos, nº 35, 28029 Madrid, Spain; 3 Carlos III Health Institute, National School of Health, C/Sinesio Delgado, nº8, 28029 Madrid, Spain; 4 Service of Preventive Medicine. Hospital Central de la Cruz Roja San José y Santa Adela, Madrid, Spain

**Keywords:** HIV testing, Preferences, Testing services

## Abstract

**Background:**

In the current context of diversity and coexistence of HIV testing approaches, limited information exists on test recipient’s views of HIV testing services and programme attributes that could ease the testing process and make it more appealing for at risk individuals who don’t know their HIV status. This study analyzed ratings given to different testing sites and programme characteristics that might facilitate testing.

**Methods:**

We analyzed data from 3120 persons attending a mobile HIV testing programme located on a central street in the gay district of Madrid.

**Results:**

64% were men (of which, 55% had had sex with other men), 59.5% were <30 years, 35.4% foreigners, 50.6% had a university degree,71.7% a regular employment, 59.3% reported multiple partners and inconsistent condom use and 56.5% had been tested for HIV. Non Governmental Organizations and specific HIV/STI centres received the maximum rating from over 60% of participants, followed by self-testing (38.9%). Pharmacies (20.8%) and hospital emergency departments (14.2%) were the worst valued testing sites. Over 80% gave the highest rating to having immediate test results, not needing a previous appointment, and free testing, while less than 50% gave the maximum rating to privacy and anonymity.

**Conclusions:**

HIV testing services that don’t require an appointment, based on free tests with rapid results are most valued by a young, not socially marginalized but high risk sexual exposure population. On the contrary, issues traditionally highly valued by health care providers or AIDS social organizations (privacy and anonymity) are much less valued.

## Background

One of the main challenges faced by high income countries in controlling the HIV epidemic is promoting early diagnosis [1-4]. Knowledge of the barriers and facilitating factors for HIV testing and counseling is essential for the design of effective interventions. Most of the studies have shown that low risk perception and fear of possible social and individual consequences derived from a positive result hinder HIV testing. Factors related to health providers, such as having to ask for an appointment, having to give personal data or having to wait for the results are all barriers that deter individuals from going to services to get tested [5]. Difficulties to access traditional health services have also been pointed, especially within most at risk and vulnerable populations [6-8]. In recent years, there has been a significant diversification of HIV counselling and testing alternatives to increase and improve the access of HIV testing in these population groups. Programmes offering rapid HIV testing in outreach and community settings are one of the strategies that have been developed to overcome some logistical obstacles associated with traditional healthcare settings [9-11] such as the amount of time spent in the testing process or having to ask for an appointment. In Spain, three medical visits are needed in order to determine a patients HIV status: 1) In the first one, either the patient asks for it or the physician recommends it; 2) a subsequent visit is scheduled to draw a venous blood sample (more invasive than rapid tests); and finally, 3) around 8 days later, the patient returns for the test results.

The few studies analyzing HIV testing preferences from the participant’s perspective were conducted before consolidation of the community based testing strategies, mainly in the United States, whereas no information is available from countries with different health systems like those in the European Union [5,12-15]. Additionally, the acceptability of programmes offering rapid HIV testing in pharmacies has never been examined. This community service is particularly accessible and constitutes another innovative alternative to improve access to testing for people who have little contact with the health system [16,17]. Identifying the services, factors and attributes that facilitate the decision to obtain a test for HIV could help remove the barriers to testing, increase testing coverage in persons at risk and target people with unrecognized HIV infection.

In the current context of multiple testing strategies, we analyzed how different testing services were rated, as well as the programme characteristics that facilitate testing in the opinion of persons attending a mobile rapid testing programme located on a central street on the limits of the gay district of Madrid.

## Methods

Between May and December of 2008, 3120 persons underwent rapid testing in a street based outreach programmme implemented by the non-governmental organisation “Madrid Positivo”. The programme was carried out in a mobile unit located on a central street frequented by young people and situated on the limits of “Chueca” known to be the gay neighbourhood of Madrid.

Those interested in getting tested, approached the mobile unit where a social educator explained what the rapid test is, how it is carried out, and its limitations in terms of results. For those who decided to get tested, healthcare staff conducted a pre-counseling session and, after obtaining signed informed consent, took a blood sample by finger prick. While awaiting the result, participants completed a self-administered anonymous questionnaire with questions on sociodemographic characteristics, sexual behaviours and on several aspects surrounding HIV testing. This last section included a list of six testing sites (see Additional file 1: Table S1) and another one listing five characteristics that might facilitate taking the test. Participants were asked to rate on a Likert scale their personal preference for each of the six testing sites (0 = Not at all preferred – 5 strongly preferred) and the importance given to the five characteristics listed (0 = Not at all important – 5 = Very important).

The question on testing services was included in November when the study had already started; therefore only 1155 persons participated, versus 2943 who answered the questionnaire on testing characteristics. There were no differences between the two groups.

Sociodemographic and behavioural characteristics of participants are described, with the sample stratified by sex and sexual behaviour: men who have sex with men (MSM), men who have sex exclusively with women (MSW), and women. The percentage of persons who gave the maximum rating for their preference for each site and the importance of each testing characteristic were calculated, and the differences were analyzed using the χ2 test. This study was approved by the institutional review board of the Carlos III Health Institute. All participants provided written informed consent.

## Results

Some 35.2% (n = 1100) were MSM, 28.7% were MSW (n = 897), and 36% (n = 1123) were women (Table 1). About 59.5% (n = 1794) were under 30 years old, and women were younger than men. Those born outside of Spain comprised 35.4% (n = 1085) of the sample and were mostly from Latin America. Over half (50.6%, n = 1577) had university education, and this percentage was lower in MSW. Most participants were single, resided in Madrid, and had regular (with contract) employment. Some 14.7% (n = 145) of MSM had ever been paid for sex versus 5.5% (n = 46) of MSW and 6.0% (n = 65) of women. Reporting two or more sexual partners without consistent condom use was more frequent in MSM (66.1%, n = 652) than in MSW (58.5%, n = 488) and women (53.3%, n = 540). However, the proportion of those who had paid for sex was twice as high in MSW (55.6%, n = 476) than in MSM (27%, n = 272), versus only 1.9% (n = 21) of women. Almost half (48%, n = 480) of MSM had been diagnosed with an STI (17.2% (n = 131) in MSW and 20.7% (n = 206) in women). Only 2.6%(n = 78) had ever injected drugs. About 74.3% (n = 783) of MSM had previously been tested versus 44.5% (n = 380) of MSW and 43.8% (n = 480) of women. Overall, 56.7% (n = 1710) took the test because they happened to pass by the mobile unit (Table 1).

**Table 1:** Sociodemographic characteristics and behavioral risk factors in people receiving rapid HIV testing in a mobile program (Madrid, 2008)

	**Men (N = 1997)**					**Women (N = 1123)**			**Total (N = 3120)**	
	**MSM**		**MSW**		**(MSM vs MSW)**			**(MSW vs women)**		
	**(N = 1100)**		**(N = 897)**							
	**N**	**%**	**N**	**%**	**p**	**N**	**%**	**p**	**N**	**%**
**Age group (years)**					0.149			0.000		
<25	300	28.4	215	25.1		400	37.2		919	30.5
25-29	278	26.3	254	29.6		335	31.2		875	29.0
≥30	480	45.4	390	45.4		339	31.6		1220	40.5
**Country of birth**					0.472			0.059		
Spain	712	66.2	575	65.2		688	63.3		1984	64.6
Other developed countries^a^	62	5.8	51	5.8		71	6.5		185	6.0
Latin America	276	25.7	225	25.5		306	28.2		818	26.7
Other developing countries	25	2.3	31	3.5		22	2.0		82	2.7
**Educational level**					0.002			0.000		
Primary	177	16.2	175	19.7		158	14.2		520	16.7
Secondary	345	31.7	322	36.3		344	30.9		1020	32.7
University	568	52.1	391	44.0		612	54.9		1577	50.6
**Marital status**					0.000			0.000		
Married	75	6.9	103	11.6		73	6.5		253	8.1
Not married	1015	93.1	788	88.4		1045	93.4		2870	91.9
**Resident in Madrid (last 12 months)**	783	72.0	667	75.1	0.000	831	74.4	0.014	2299	73.7
**Employment status (last 12 months)**					0.260			0.010		
Regular employment	808	74.5	649	73.2		755	68.1		2227	71.7
No regular employment	134	12.4	134	15.1		162	14.6		433	13.9
Other	142	13.1	104	11.7		192	17.3		445	14.3
**≥2 sexual partners and inconsistent condom use (last 12 months)**	652	66.1	488	58.5	0.000	540	53.3	0.470	1680	59.3
**Ever been paid for sex**	145	14.7	46	5.5	0.000	65	6.0	0.726	257	8.8
**Ever paid for sex**	272	27.0	476	55.6	0.000	21	1.9	0.000	775	26.0
**Reported STI (lifetime)**	480	48.0	131	17.2	0.000	206	20.7	0.126	822	29.8
**Ever injected drugs**	29	2.8	27	3.3	0.518	21	1.9	0.051	78	2.6
**Previous HIV testing**	783	74.3	380	44.5	0.000	480	43.8	0.644	1653	54.8
**Reason for having the test in this program**					0.375			0.021		
Knew about the program	443	41.8	367	43.3		419	38.4		1235	41.0
Passed by and saw the program	597	56.4	455	53.7		648	59.4		1710	56.7
Other reason	19	1.8	26	3.1		24	2.2		69	2.3

Note. *MSM* Men who have sex with men, *MSW* Men who have sex exclusively with women, *STI* Sexually transmitted infection.

^a^Western Europe, North America, Australia, Japan.

The two services for which the largest percentage of participants assigned the highest preference rating were non-governmental organizations (NGOs) (62.3%, n = 650) and HIV/STI testing centres (60.8%, n = 618), followed by home self-testing, (although it is not an available option) (38.9%, n = 373). Primary care doctors (28.2%, n = 289), pharmacies (20.8%, n = 198), and hospital emergency services (14.2%, n = 134) were the options chosen by the fewest (Table 2). This pattern was maintained for all subgroups, although different variables were associated with a higher preference depending on the testing site. The most important differences were that preference for an NGO or home self-testing increased with age, and that Latin Americans, those with primary education, and those who did not previously know about the programme more often preferred the primary care doctor.

**Table 2:** Ratings given to HIV testing services and program characteristics by people receiving rapid HIV testing in a mobile program (Madrid, 2008)

	**Preferences for HIV testing services (N=1155)**^**a**^						**Importance of HIV test characteristics (N=2943)**^**a**^				
	**Non-governmental organization**	**HIV/STI centre**	**Self-testing at home**	**Primary care doctor**	**Pharmacy**	**Hospital emergency department**	**Immediate results**	**Free of charge**	**Appointment not needed**	**Private (no one knows me)**	**Anonymous (no identification required)**
	**(N=1043)**^**b**^	**(N=1016)**^**b**^	**(N=960)**^**b**^	**(N=1024)**^**b**^	**(N=951)**^**b**^	**(N=944)**^**b**^	**(N=2891)**^**b**^	**(N=2786)**^**b**^	**(N=2763)**^**b**^	**(N=2726)**^**b**^	**(N=2722)**^**b**^
	**%**	**%**	**%**	**%**	**%**	**%**	**%**	**%**	**%**	**%**	**%**
**0=Not at all**	2.1	3.4	37.1	20.1	30.2	27.4	0.9	2.5	1.1	13.5	11.3
**1**	1.2	2.9	7.0	10.5	16.1	16.9	0.6	1.2	0.6	5.1	4.5
**2**	3.8	3.9	3.9	10.7	11.0	14.5	1.5	3.1	1.6	9.0	7.8
**3**	10.1	12.9	5.6	17.8	12.5	15.9	4.2	5.5	5.2	14.2	13.8
**4**	20.4	16.0	7.6	12.6	9.4	11.0	8.1	6.5	10.7	12.7	14.0
**5=Strongly/Very**	62.3	60.8	38.9	28.2	20.8	14.2	84.8	81.3	80.8	45.5	48.7

^a^Number of people who could answer this question; ^b^Number of persons who actually answered each item.

No differences were observed in the ranking of most preferred services between persons with and without previous testing experience. Those who already knew about the programme were slightly more favourable to NGOs and home self testing and those who stated having discovered it because they were in the area and happened to see the service, gave better ratings to traditional settings such as primary care or emergency departments (Table 3).

**Table 3:** HIV testing preferences in people receiving rapid HIV testing in a mobile program, by sociodemographic characteristics and behavioral risk factors (Madrid, 2008)

	**HIV testing services(N=1155)**^**a**^						**HIV test characteristics (N=2943)**^**a**^				
	**Non-governmental organization**	**HIV/STI centre**	**Self-testing at home**	**Primary care doctor**	**Pharmacy**	**Hospital emergency department**	**Immediate results**	**Free of charge**	**Appointment not needed**	**Private (no one knows me)**	**Anonymous (no identification required)**
	**(N=1043)**^**b**^	**(N=1016)**^**b**^	**(N=960)**^**b**^	**(N=1024)**^**b**^	**(N=951)**^**b**^	**(N=944)**^**b**^	**(N=2891)**^**b**^	**(N=2786)**^**b**^	**(N=2763)**^**b**^	**(N=2726)**^**b**^	**(N=2722)**^**b**^
	**%**	**%**	**%**	**%**	**%**	**%**	**%**	**%**	**%**	**%**	**%**
**All**	62.3	60.8	38.9	28.2	20.8	14.2	84.8	81.3	80.8	48.7	45.5
**Age group (years)**	p=0.000	p=0.091	p=0.010	p=0.780	p=0.018	p=0.669	p=0.000	p=0.023	p=0.219	p=0.009	p=0.001
<25	49.8	56.4	31.2	28.3	14.4	15.4	80.0	83.1	78.7	44.6	40.1
25- 29	64.2	65.2	39.4	26.6	23.7	13.4	86.2	82.3	81.0	48.5	46.4
≥30	69.0	61.9	43.1	29.0	21.7	13.0	87.6	78.5	81.8	51.8	48.6
**Country of birth**	p=0.980	p=0.034	p=0.189	p=0.000	p=0.159	p=0.899	p=0.821	p=0.000	p=0.559	p=0.463	p=0.967
Spain and other developed countries^c^	62.2	63.1	40.2	23.7	21.7	13.9	84.8	79.6	80.3	49.3	45.5
Latin America	62.2	53.0	35.1	43.1	16.1	13.6	85.0	87.5	82.2	46.4	44.9
Other developing countries	60.0	57.9	23.5	22.2	11.1	17.6	81.8	73.1	81.6	49.0	44.7
**Educational level**	p=0.162	p=0.827	p=0.172	p=0.035	p=0.195	p=0.345	p=0.980	p=0.000	p=0.265	p=0.460	p=0.630
Primary	54.9	57.4	33.3	37.2	20.7	16.2	84.4	88.4	83.3	47.3	44.6
Secondary	62.2	60.5	38.3	28.0	17.9	16.1	85.0	83.2	80.8	50.5	46.7
University	64.7	61.7	41.5	26.0	22.9	12.9	84.9	78.1	80.0	48.3	45.1
**Gender/Sexual behaviour**	p=0.082	p=0.019	p=0.366	p=0.183	p=0.760	p=0.830	p=0.141	p=0.256	p=0.220	p=0.096	p=0.234
MSM	63.6	64.0	41.9	24.5	20.3	13.0	85.5	81.3	80.6	46.2	47.3
MSW	57.4	54.8	36.9	31.1	22.1	14.7	82.8	79.4	79.1	49.3	43.0
Women	65.4	63.7	38.2	28.7	20.0	14.8	85.9	82.5	82.4	51.1	45.4
**Ever been paid for sex**	p=0.498	p=0.893	p=0.534	p=0.105	p=0.030	p=0.139	p=0.847	p=0.049	p=0.370	p=0.195	p=0.067
Yes	57.7	59.6	34.8	38.0	7.3	6.7	85.0	86.6	83.2	53.0	51.6
No	62.4	60.6	39.3	27.4	21.4	14.6	84.5	80.8	80.4	48.0	44.5
**Ever paid for sex**	p=0.716	p=0.960	0.923	p=0.308	p=0.618	p=0.676	p=0.849	p=0.150	p=0.076	p=0.350	p=0.841
Yes	62.8	60.7	39.2	30.4	22.0	13.4	84.9	79.2	78.4	50.1	45.5
No	61.5	60.8	38.7	27.0	20.4	14.5	84.6	81.6	81.4	47.8	44.9
**Reported STI (lifetime)**	p=0.067	p=0.244	p=0.190	p=0.074	p=0.531	p=0.034	p=0.165	p=0.025	p=0.029	p=0.458	p=0.207
Yes	66.9	63.6	42.6	32.0	21.6	18.8	86.1	83.6	83.5	48.0	46.8
No	60.5	59.4	37.7	26.1	19.6	13.1	84.0	79.8	79.7	49.1	44.1
**Previous HIV test**	p=0.214	p=0.527	p=0.065	p=0.053	p=0.053	p=0.449	p=0.144	p=0.028	p=0.023	p=0.003	p=0.819
Yes	64.1	59.8	36.1	30.5	18.2	14.8	85.7	82.7	82.3	46.1	45.7
No	60.3	61.7	42.0	25.0	23.4	13.1	83.8	79.4	78.8	51.9	45.2
**Reason for testing in this program**	p=0.050	p=0.461	p=0.144	p=0.001	p=0.774	p=0.005	p=0.000	p=0.000	p=0.134	p=0.001	p=0.004
Knew about the program	65.7	61.7	41.7	23.6	20.3	11.2	88.5	79.0	82.3	52.9	49.1
Passed by and saw it	58.4	60.9	35.6	34.9	21.5	18.2	82.2	83.4	79.8	45.7	42.8

Note.Percentages refer to the proportion of people who gave the maximum rating for each item.

Note. *MSM* Men who have sex with men, *MSW* Men who have sex exclusively with women, *STI* Sexually transmitted infection.

^a^ Number of people who could answer this question; ^b^Number of persons who actually answered each item.

^c^Western Europe, North America, Australia, Japan.

Over 4 out of 5 participants gave the maximum rating for the importance of three testing characteristics: having immediate results (84.8%, n = 2452), free testing (81.3%, n = 2265), and testing without previous appointment (80.8%, n = 2233). However, less than half of participants gave this rating for “being tested in a place where nobody knows you” (48.7%, n = 1326) or not needing to identify themselves (45.5%, n = 1240) (Table 2).

Notable in the bivariate analysis is the greater importance that Latin Americans and those without university education give to free testing, and that immediate test results, not having to identify oneself, and going to a centre where one is not known become more important with increasing age.

Participants who had already been tested in the past, placed a higher value on not having to ask for an appointment than those with no previous testing experience who gave slightly more importance to being able to go to a service where nobody knew them. Those who already knew the programme gave slightly more importance to receiving the test results immediately and to having the possibility of going to services where nobody knew them (Table 3).

## Discussion

Until now, mobile services in the streets are being considered as a good strategy to promote HIV testing only in socially marginalized populations [9,18]. However, this outreach mobile programme attracted a non-socially marginalized population, mainly comprised by young people, with high educational level, regular employment and with high levels of sexual risk exposures to HIV. An important proportion, especially within MSM, had previously accessed other services to take the HIV test.

This is the first European based study that identifies the preferences for testing services and factors that could facilitate testing from the client’s perspective. In the US, recommendations for expanding HIV testing outside medical settings were published back in 2003 [19] and CDC revised recommendations for performing routine HIV screening in all health care settings three years later [1]. In Europe, efforts towards expanding HIV testing to a wide variety of healthcare and nonclinical community services has been more recent [4] and focused mainly on the most at risk populations, although the UK and France have proposed population based screening strategies [20-22].

In this context of technological advances and plurality of testing scenarios, most users in our study, as well as the subgroups studied, clearly opt for specific services for HIV diagnosis frequented primarily by the most at-risk populations, like those operated by NGOs and centres for HIV/STI diagnosis.

It is also notable that interest in home self-testing was considerably higher than described to date [14,15,23], even though this option is not available in Spain, and is still not object of intense public debate. Conversely, the most traditional health-care setting, the primary care doctor, was rated similar to self-testing only among Latin American immigrants and persons with lower educational level. Pharmacies obtained high ratings from only 1 out of 5 participants. This highly accessible setting has not been evaluated in other countries. The advisability of using pharmacies for HIV testing has been discussed in Spain, and two pilot programmes in Catalonia [17] and the Basque country were launched in 2009 [16]. In the study conducted in the Basque country, 55% of those who underwent rapid testing stated convenience and accessibility as the two most important reasons for choosing this particular setting to get tested [16].

Hospital emergency departments received the maximum preference rating from the smallest proportion of participants. Unlike in the United States, these sites are not routinely used for diagnostic purposes in Spain. In addition, some studies are questioning the effectiveness of the non-targeted HIV screening in this setting because of its modest public health impact [24,25]. In our study, having previous testing experience had no influence in the rating given to the different services. While people who had been tested previously gave their ratings based on their own experience, those with no prior testing experience could have rated the different services basing their opinion on the views and experiences concerning testing of their social circle.

With regard to programme features that may facilitate HIV testing, this study shows that aspects like privacy or anonymity, cited in other studies as very important [8], are less important for this population than other characteristics of rapid testing programmes like being free of charge (shared with most programmes), not having to request an appointment (characteristic of some programmes), or knowing the result immediately (a distinctive feature of these programmes). It is possible that privacy and anonymity have been less valued because participants consider they are guaranteed rights and therefore take them for granted. However, it is important to note that the three most valued aspects are not exclusive characteristics of this street-based HIV rapid testing programme. In Spain, HIV testing is offered free of cost at all levels of the national public health system and in some cities, there are specific HIV/STI clinics that offer the test without the need of a previous appointment. In addition, programmes offering rapid testing in a wide range of settings have widely spread in recent years.

Other studies have also clearly identified the importance given by clients to factors such as being able to choose the type of HIV test, having it free of cost and receiving the test result in the same visit [26-28].

In our study, individuals born in Latin America and those with lower educational level (characteristics associated with less favored socioeconomic conditions), are the groups that value gratuity the most. Several studies point out that preoccupation of being seen entering a sexual health clinic by members of their own community network or running into someone they already know are both barriers to HIV testing in migrants and ethnic minorities [29-31]. However, “being tested in a place where nobody knows you” is equally valued by Spaniards and immigrants. Similarly, no differences were found between these two groups when they were asked about the importance of getting tested anonymously, despite fear that disclosure of HIV status could affect the permit application process in undocumented immigrant’s residency [32]. In a similar way to the rating given to services, previous testing experience had little influence on the ranking of the factors that could facilitate the test.

Interpretation of these results must take into account that they obviously depend on the characteristics of the setting were the sample was recruited. Generalizations can not be made to other populations recruited in different settings such as indoor NGO venues or formal health facilities. Opinions towards NGOs and rapid testing might be biased as participants voluntarily attended a programme which fits in the NGO category and uses rapid tests. However, it is important to underscore that the opinions of those who already knew about the service were quite similar to the ones referred by those who discovered the mobile unit when passing by.

Respondents were asked about self-testing at home, an option they had not actually experienced. However, in a Spanish study that evaluated the feasibility of self-performing a rapid test and interpreting the results, most participants (83.9%) were more motivated to use this testing option, after having carried out self-testing [33].

Our data are based on self-report and could be affected by lack of sincerity in the responses and by social desirability bias. However, the use of an anonymous and self-administered questionnaire may have helped to obtain more complete and sincere self-reports in those sensitive aspects of the survey. The non-response on questionnaire items (ranging from 1.8% to 18.3%) may partly be due to the use of a self-completed questionnaire. Since the highest percentage was found for the least known settings, it is logical to assume that this corresponds to the option “don’t know/no answer,” a response category that was not included to simplify the process. Accordingly, the real differences are likely to be higher than those described.

## Conclusions

HIV testing services that do not require an appointment, based on free, non-invasive tests with rapid results and carried out by health educators, healthcare personnel, or even by individuals themselves, could promote demand and improve access to testing in young people with high levels of sexual risk exposure to HIV, particularly in especially vulnerable populations like MSM or immigrants.

## Competing interests

The authors declare that they have no competing interests.

## Authors’ contributions

LF and MJB conceived and designed the study, JH, SFB, MERS and JP participated in the statistical analysis, MJB and JH wrote the manuscript, SFB, MERS, JP drafted the manuscript. All authors have read and approved the manuscript.

## Pre-publication history

The pre-publication history for this paper can be accessed here:

http://www.biomedcentral.com/1471-2458/13/791/prepub

## Supplementary Material

Additional file 1: Table S1 “General characteristics of HIV testing sites addresed in the study”. Click here for file
