# Supplementary material for: Preferred HIV testing services and programme characteristics among clients of a rapid HIV testing programme
Source: BMC Public Health. 2013 Aug 30;13:791. doi: 10.1186/1471-2458-13-791 (PMC3765864; doi:10.1186/1471-2458-13-791)
Supplement: Additional file 1: Table S1 — “General characteristics of HIV testing sites addresed in the study”. [file 1471-2458-13-791-S1.doc]

**TableS1: General characteristics of HIV testing sites addressed in the study.**

| **Primary Care Doctor** | During the data collection period, (2008) primary care could be accessed without restrictions both by Spaniards and immigrants (regardless of legal status).  In the Spanish public primary care network, there is no routine screening strategy. Testing is only carried out when demanded by the patient or when proposed by the physician.  Testing is free and confidential and, generally, conventional tests are used. The average waiting time for the result is approximately 8 days.  In some cities there are some programmes in which HIV rapid testing is offered.  Especially in large urban areas there is also private practice and, increasingly, private insurances are also offering primary care within their range of services. |
| --- | --- |
| **Hospital Emergency Department** | Most hospitals, especially in small towns, belong to the national health system. In emergency departments, assistance is guaranteed regardless of nationality and legal situation.  Currently in Spain, HIV testing is not routinely carried out except in very particular situations and depends on the protocols established by each hospital. |
| **HIV/STI Centres** | Most of them belong to the National Health System. They are usually located in large urban centers and offer counseling and conventional blood testing for HIV and also diagnose and treat other sexually transmitted infections.  Testing is voluntary, free, anonymous, and confidential and generally does not require a previous appointment.  However, there is also a wide range of small private clinics on which conditions change significantly from one another. |
| **Non-governmental Organization** | Currently, the set of NGOs that offer HIV testing is very wide and heterogeneous in terms of schedules, geographic location and supplemental services they offer. Some are aimed at particularly vulnerable populations (injecting drug users, people who engage in prostitution, men who have sex with men, migrants, etc.) while others don’t target a specific population, testing anyone who requests it.    Most of these services are located indoors, at the premises of the NGOs, and some have developed outreach programs.  Rapid testing is always used, but while some use oral fluid based tests, others use -blood based kits.  HIV testing is anonymous, confidential and offered free of cost.  Some require making an appointment due to long and structured counseling sessions, regardless of the test result. Others, work under a first-come, first-served basis as their counseling sessions are very brief in the case of a negative result. |
| **Pharmacy** | Some Spanish regions are offering HIV rapid testing at certain pharmacies. However, this is not a possibility extended neither to the whole national territory nor to all pharmacies within those regions in which this option exists.  Each program has its own characteristics when talking about accessibility, times and prices, anonymity, etc.  Testing takes place in a space where privacy and confidentiality can be assured and where personalized attention can be delivered. |
| **Self-testing at home** | In Spain, sale and distribution of this testing option is not authorized. However, self-test kits are easily available for purchase online.  It is currently being object of an intense scientific debate; therefore we decided to include it in the study. |
